# A Valveless Pulsatile Pump for Heart Failure with Preserved Ejection Fraction: Hemo- and Fluid Dynamic Feasibility

**DOI:** 10.1007/s10439-020-02492-2

**Published:** 2020-03-30

**Authors:** Andreas Escher, Young Choi, Fraser Callaghan, Bente Thamsen, Ulrich Kertzscher, Martin Schweiger, Michael Hübler, Marcus Granegger

**Affiliations:** 1 Pediatric Cardiovascular Surgery, Department of Surgery, Pediatric Heart Center, University Children’s Hospital Zurich, Zurich, Switzerland; 2 Children’s Research Center, University Children’s Hospital Zurich, Zurich, Switzerland; 3 Center for MR Research, University Children’s Hospital Zurich, Zurich, Switzerland; 4 Biofluid Mechanics Laboratory, Institute for Imaging Science and Computational Modelling in Cardiovascular Medicine, Charité-Universitätsmedizin Berlin, Augustenburger Platz 1, Berlin, Germany; 5 Department of Mechanical and Process Engineering, Institute for Mechanical Systems, ETH Zurich, Zurich, Switzerland

**Keywords:** CFD, 4D-flow MRI, Single cannula, Isolated porcine heart model, *In vitro*, *Ex vivo*, *In silico*, Washout, Stagnation, Hybrid mock loop

## Abstract

Treatment of heart failure with preserved ejection fraction (HFpEF) remains a major unmet medical need. An implantable valveless pulsatile pump with a single cannula—the CoPulse pump—may provide beneficial hemodynamic support for select HFpEF patients when connected to the failing ventricle. We aimed to demonstrate hemodynamic efficacy and hemocompatible design feasibility for this novel assist device. The hemodynamic effect of the pump was investigated with an *in vitro* circulatory mock loop and an *ex vivo* isolated porcine heart model. The hydraulic design was optimized using computational fluid dynamics (CFD), and validated by 4D-flow magnetic resonance imaging (MRI). The pump reduced left atrial pressure (> 27%) and increased cardiac output (> 14%) *in vitro*. *Ex vivo* experiments revealed elevated total stroke volume at increased end-systolic volume during pump support. Asymmetric cannula positioning indicated superior washout, decreased stagnation (8.06 mm^2^ vs. 31.42 mm^2^), and marginal blood trauma potential with moderate shear stresses (< 24 Pa) *in silico*. Good agreement in flow velocities was evident among CFD and 4D-flow MRI data (*r* > 0.76). The CoPulse pump proved hemodynamically effective. Hemocompatibility metrics were comparable to those of a previously reported, typical pulsatile pump with two cannulae. The encouraging *in vitro, ex vivo,* and hemocompatibility results substantiate further development of the CoPulse pump.

**Electronic supplementary material:**

The online version of this article (10.1007/s10439-020-02492-2) contains supplementary material, which is available to authorized users.

## Introduction

Heart failure (HF) can be classified into three categories based on the ejection fraction (EF): heart failure with reduced (HFrEF), mid-range (HFmrEF), and preserved ejection fraction (HFpEF). While HFrEF has attracted most of the scientific and clinical research, difficulties of diagnosing the typical manifestations of HFpEF (e.g. impaired diastolic ventricular function) have left this phenotype less clearly understood.^17^,^23^

HFpEF accounts for roughly half of all heart failure cases^24^ and constitutes a patient population which is projected to grow.^23^ Furthermore, HFpEF exhibits similar incidence of mortality and morbidity as HFrEF.^2^,^23^,^24^,^26^ An unaddressed clinical need for effective treatment of HFpEF exists as there are no clinically available options that significantly improve outcomes: Pharmacologic studies have shown that typical heart failure medications are ineffective in HFpEF patients.^4^,^20^ Further, intra-arterial shunt devices, specifically aiming to reduce the commonly elevated left atrial pressure (LAP) among HFpEF patients, have not yet shown significant efficacy.^27^

Mechanical circulatory support (MCS) provides an alternative means for reducing LAP. MCS has been studied in patients with hypertrophic and restrictive cardiomyopathies with low ejection fraction,^25^ but has not yet been clinically evaluated in HFpEF patients. However, numerical studies have shown that MCS, through either ventricular or atrial cannulation, could be an effective treatment strategy for certain phenotypes of HFpEF.^5^,^22^ Yet, contemporary MCS systems are accompanied by severe adverse events related to device hemocompatibility. Thromboembolic and bleeding events are stirred by the non-physiologic flow within the device—high shear rates and prolonged residence times of blood particles lead to blood trauma and thrombus formation.^3^ In pulsatile pumps, the valvular regions are an additional source for thrombus formation.^14^

In a recent numerical study, we showed the potential of using a 30 mL volume displacement pump with a single valveless cannula directly connected to the diseased ventricle to improve the hemodynamic state of HFpEF patients.^11^ By synchronously filling and emptying the pump with the diseased left ventricle (LV), the pump could provide additional stroke volume (SV) by capitalizing on the stiff systolic properties of the LV during ejection. Additionally, the end-systolic volume could be increased, thereby potentially mitigating the risk of suction events. This novel concept of an assist device for HFpEF—henceforth denoted as CoPulse pump—may additionally address some of the hemocompatible design weaknesses associated with conventional MCS: (1) pulsatile devices have been associated with lower shear rates than rotary blood pumps,^7^ (2) the valveless design eliminates the main location of blood clots for pulsatile devices.^14^

The present study aimed at continuing the development of the CoPulse pump to a functioning prototype. In accordance with the state-of-the-art development practice for ventricular assist devices (VADs), this involved fabrication of the physical pump system, validation of earlier *in silico* hemodynamic findings^11^*in vitro*, further evaluation of the CoPulse efficacy *ex vivo*, and preliminary optimization of the pump’s hydraulic characteristics using computational fluid dynamics (CFD) and 4D-flow magnetic resonance imaging (MRI).

## Materials and Methods

### The CoPulse Pump—Prototype Fabrication and Actuation

**Figure 1:**
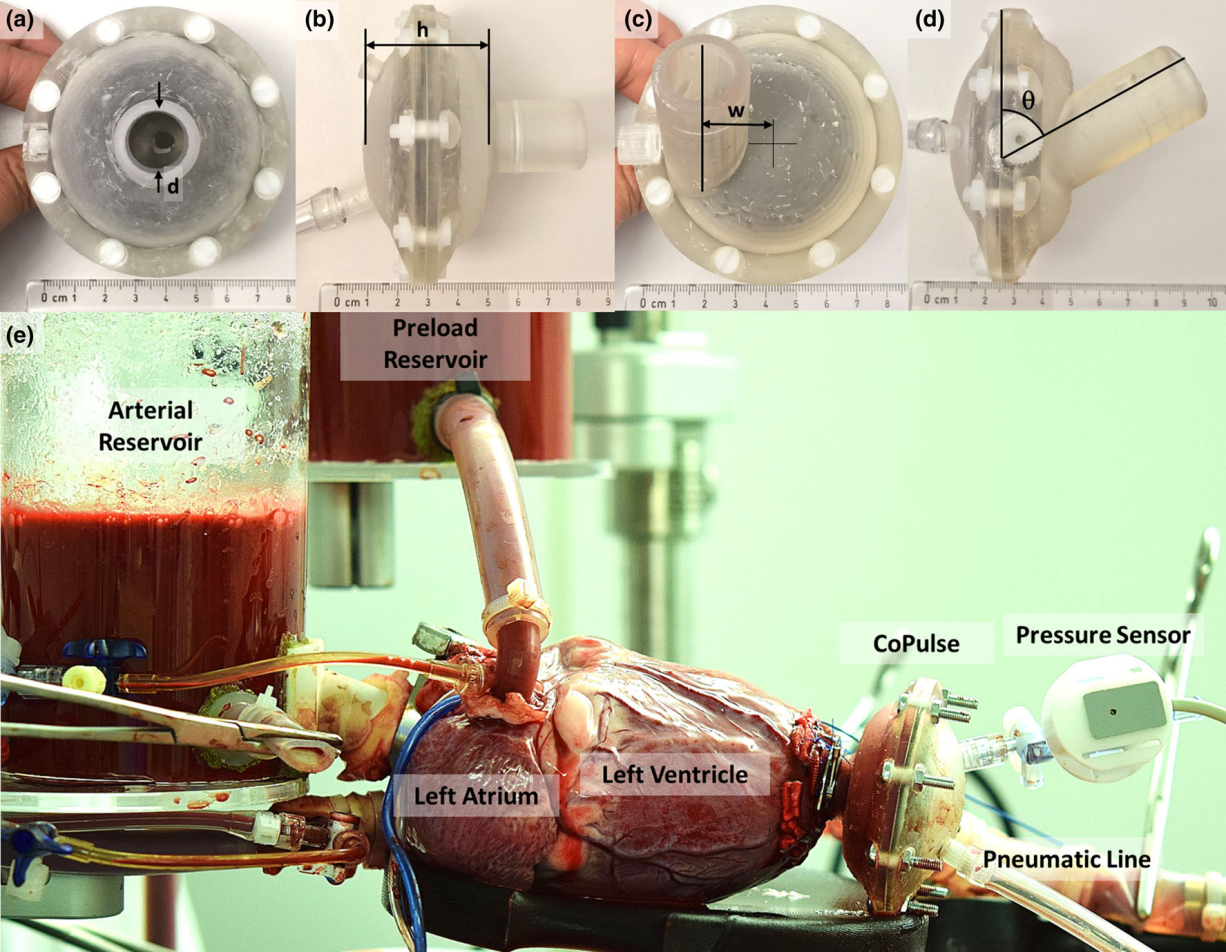
(a) Top-view photo of the symmetric CoPulse prototype, (b) Side-view photo of the symmetric CoPulse prototype, (c) Top-view photo of the asymmetric CoPulse prototype, (d) Side-view photo of the asymmetric CoPulse prototype, (e) Photo of the CoPulse pump implanted into the left ventricle of the *ex vivo* isolated porcine heart. (a)–(d) label the design variables used for geometric pump optimization and are denoted by *h* (pump chamber height), *d* (cannula diameter), *w* (cannula offset distance), and *θ* (cannula orientation). The geometric range of the respective design variables is reported in Supplementary Table 1. (a)–(d) also show the pump assembled without any metallic parts allowing for 4D-flow MRI testing.

Functional prototypes of the CoPulse pump were fabricated to be used for all experimental investigations and according to the symmetric (Figs. 1a and 1b) and the asymmetric (Figs. 1c and 1d) designs that were hydraulically optimized using CFD (Fig. 4). The pump housing was 3D-printed with a Formlabs Form 2 using Clear Resin (Formlabs, Somerville, MA, USA) with minimal wall thickness of 2.5 mm. The membrane (arc diameter: 53 mm, amplitude: 12.6 mm) was acquired from a BerlinHeart Excor pump (BerlinHeart GmbH, Berlin, Germany) and divided the pump into a pneumatic and a blood chamber to displace an approximate 30 mL stroke volume (Supplementary Figure 1). A single valveless cannula (inner diameter: 15 mm, length: 25 mm) provided connection of the blood chamber to the LV apex (Fig. 1e).

**Figure 2:**
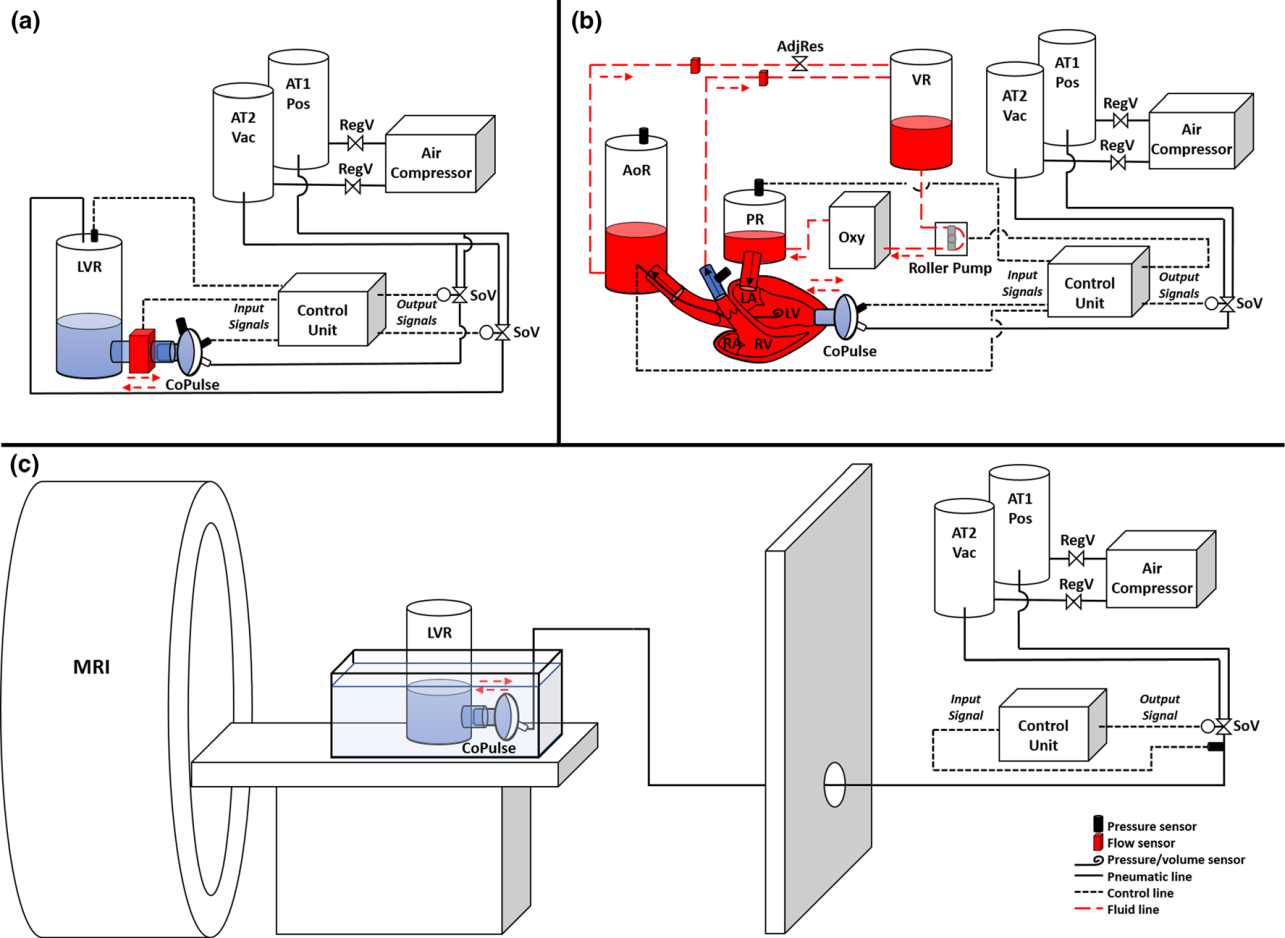
Schematic diagrams of (a) the *in vitro* hybrid mock circulatory loop system, (b) the *ex vivo* isolated beating heart setup, (c) the 4D-flow MRI setup. Air tank 1 for positive pressure (AT1 Pos); air tank 2 for vacuum pressure (AT2 Vac); regulator valve (RegV); solenoid valve (SoV); left ventricular reservoir (LVR); aortic reservoir (AoR); preload reservoir (PR); venous reservoir (VR); oxygenator (Oxy); adjustable resistance clamp (AdjRes); left atrium (LA); left ventricle (LV); right atrium (RA); right ventricle (RV).

A pneumatic line connected the pneumatic chamber of the pump to a pneumatic system, which transferred positive and vacuum pressures to drive the reciprocating membrane movement. The pneumatic system (Fig. 2) consisted of an air compressor (KNF Neuberger PM21308-023.1.2, Freiburg, Germany), two air tanks—one for positive pressure and another for vacuum pressure, relief valves (Niezgodka GmbH 91-2508-4 and 1831, Hamburg, Germany), and a 5/3-way solenoid control valve (Festo MPYe-5-1/4-010-B, Esslingen am Neckar, Germany). To synchronize ejection and filling of the CoPulse pump with the LV cardiac cycle, a control system was developed that utilized the left ventricular pressure (LVP) as an input signal; the control system and pump phasing are described in detail in Supplementary Figure 2. The control system was implemented in MATLAB Simulink (The MathWorks, Natick, MA, USA) and deployed onto a dSPACE MicroLAB Box (dSPACE GmBH, Paderborn, Germany).

### Validation of Hemodynamic Efficacy

#### *In Vitro* Analysis in a Hybrid Mock Circulation

The pump prototype was tested *in vitro* in a hybrid mock circulatory loop to evaluate pump and control system functionality, and to validate hemodynamic findings from previous *in silico* studies^11^ (Fig. 2a). Unlike the *in silico* studies, the hybrid mock circulatory loop provided a platform to test the functionality of the CoPulse pump and to model the cardiovascular system’s response to a physical prototype of the pump. The loop consisted of three main components: (1) the hydraulic loop, (2) the pneumatic actuation system, and (3) the software including the control unit running a cardiovascular numerical model^11^ as well as the control algorithm for the CoPulse pump. The hydraulic loop consisted of the LV reservoir (LVR), the CoPulse pump, and a cannula that connected them to allow for clamp-on flow measurement (Sonoflow CO.55, Sonotec GmbH, Halle, Germany; measurement error: ± .024 L/min for flows 0–1.2 L/min and ± 2% for flows > 1.2 L/min). The loop was filled with a glycerol/water mixture (3.0–3.5 mPa s). Measured pump flow (*Q*_Pump_) (example waveform shown in Supplementary Figure 2B) was passed to the numerical model to simulate the cardiovascular system’s response to CoPulse support. Briefly, the numerical model was implemented as a lumped parameter model of the cardiovascular system as presented by Granegger *et al*.^11^ Model parameters were adapted to achieve typical HFpEF conditions as previously reported.^5^,^11^,^22^ The resultant LVP output from the numerical model was passed as the setpoint to a PID control system, which in turn actuated a separate solenoid control valve to control the pressure in the LVR. Thus, the LVP was controlled to simulate the modeled LVP and was, analogous to pneumatic and hydraulic pump pressures, measured using an APT300 pressure transducer (Harvard Apparatus, Holliston, MA, USA; measurement error: ± 1.7%). All other reported flows and pressures were outputs of the numerical model^11^: LAP, cardiac output (CO), aortic pressure (AoP), pulmonary artery pressure (PAP), and left ventricular volume (LVV).

The hemodynamic effect of the pump was evaluated in four typical end-stage HFpEF phenotypes, each of which were implemented separately as part of the numerical model (Supplementary Figure 3): (I) Genetically inherited hypertrophic cardiomyopathy, (II) Infiltrative cardiomyopathy with restrictive physiology, (III) Nonhypertrophic cardiomyopathy, (IV) Normal ejection fraction with comorbidities (i.e hypertension, coronary artery disease) and hypertrophy.^5^,^11^ The hybrid model outputs for LVP, LVV, AoP, PAP, LAP, and CO were used to validate previous *in silico* results.^11^

#### *Ex Vivo* Isolated Heart Experiments

Following the *in vitro* study, the pump prototype was tested *ex vivo* in an isolated beating porcine heart setup to investigate the interaction between the cardiac mechanics of a functional left ventricle and the pump, as well as to further evaluate the hemodynamic efficacy of the pump (Figs. 1e and 2b). The previously described isolated heart setup^10^,^12^ consisted of a pressure-controlled preload reservoir (PR), an aortic reservoir (AoR), a venous reservoir (VR), an oxygenator/heat exchanger, and a controllable roller pump to set the preload. An adjustable hosecock clamp on the systemic return line between the AoR and the VR allowed for manual control of the afterload resistance. AoP, LAP, PAP, and pneumatic pump pressure were measured using APT300 pressure transducers. Aortic (AoF) and coronary flow (CoF) were recorded using Sonoflow CO.55 clamp-on flow sensors. LVP and LVV were measured using a Ventri-Cath 507 pressure-volume (PV) Loop Catheter (ADInstruments, Sydney, Australia).

All experiments were approved by the Ethics Committee (Approval No. 219/16) of the Canton of Zurich. The hearts (*n* = 4) were explanted and connected to the setup as previously described.^10^,^12^ In 3 hearts the pump was implanted successfully and maintained functionality. Constant LAP (*n* = 3) and constant CO (*n* = 3) measurements were recorded with each measurement of a single type being conducted within a separate heart. Two sets of reliable LVV data were recorded for both constant LAP and constant CO experiments.

Following completion of each experiment, the heart’s end-diastolic pressure-volume relationship (EDPVR) was determined by inflating a balloon with water inside the LV while measuring both the pressure and volume. LVV conductance catheter measurements were calibrated using the measured stroke volume to calibrate the slope factor and the EDPVR to calibrate the parallel conductance.

### Data Analysis for *In Vitro* and *Ex Vivo* Hemodynamic Evaluation

All data was recorded by the dSPACE MicroLab Box at 200 Hz and analyzed using MATLAB software. All mean hemodynamic parameter values were calculated on a beat-to-beat basis, averaged over 10 second epochs and presented as mean ± standard deviation.

To evaluate the effect of the pump on the LV stroke volume, the stroke volumes of the LV (${\text{SV}}_{\text{LV}} )$, pump (${\text{SV}}_{\text{Pump}}$), and LV + pump $( {\text{SV}}_{{{\text{LV}} + {\text{Pump}}}} )$ were calculated as:

$${\text{SV}}_{\text{LV}} = {\text{EDV}} - {\text{ESV}}$$

$${\text{SV}}_{\text{Pump}} = { \hbox{max} }\left( {\mathop \smallint \limits_{{t_{0} }}^{{t_{\text{end}} }} Q_{\text{Pump}} dt} \right) - { \hbox{min} }\left( {\mathop \smallint \limits_{{t_{0} }}^{{t_{\text{end}} }} Q_{\text{Pump}} dt} \right)$$

$${\text{SV}}_{{\text{LV}} + {\text{Pump}}} = \frac{{\text{CO}}}{{\text{HR}}}$$

where EDV is the end-diastolic volume (mL), ESV the end-systolic volume (mL), and HR the mean heart rate (bpm). The volume of the pump was derived as the cumulative numerical time integral of the pump flow. Then the stroke volume was calculated as the difference between the maximal and minimal pump volumes over a single pump cycle with *t*_0_ denoting the start of ejection and *t*_end_ the end of filling.

To evaluate how cardiac energetics are affected by the CoPulse pump, the work performed by the pump was calculated as the area enclosed by the pump fluid PV loop. Furthermore, the left ventricular stroke work (SW_LV_), potential energy (PE_LV_), and the pressure-volume area (PVA_LV_) were calculated assuming a zero pressure ventricular volume V_0_ of 0 mL, a linear end-systolic pressure-volume relationship (ESPVR), and the measured EDPVR. Pump volumetric and work efficiency were calculated as:

$${\text{Pump}}\, {\text{Volumetric }}\,{\text{Efficiency}} = \frac{{{\text{SV}}_{{{\text{LV}} + {\text{Pump, }}\,{\text{CoPulse }}\,{\text{Support}}}} - {\text{SV}}_{{{\text{LV, }}\, {\text{Baseline}}}} }}{{{\text{SV}}_{{{\text{Pump,}}\,{\text{CoPulse }}\,{\text{Support}}}} }}$$

$${\text{Pump }}\,{\text{Work}}\, {\text{Efficiency = }}\frac{{{\text{SW}}_{{{\text{LV,}}\, {\text{CoPulse }}\,{\text{Support}}}} + {\text{W}}_{{{\text{Pump,}}\, {\text{CoPulse }}\,{\text{Support}}}} - {\text{SW}}_{{{\text{LV,}}\, {\text{Baseline}}}} }}{{{\text{W}}_{{{\text{Pump,}}\,{\text{CoPulse}}\, {\text{Support}}}} }}$$

### Investigation of Fluid Dynamics and Related Hemocompatibility

#### Numerical Simulations

CFD was employed for numerical flow inspection, hemocompatibility characterization and hydraulic design optimization of the pump. The fluid volume was discretized with the CFD package Star CCM+ (Siemens, Munich, Germany), resulting in an unstructured core volume mesh composed of approximately 1.5 million polyhedral cells. Controlled local mesh refinement was combined with five prism layers at the walls to better resolve the high gradients in boundary layers.

While a wall boundary with no-slip condition was stipulated for the inner blood chamber and cannula surface, a pressure outlet boundary condition was set at the cannula inlet. The membrane was implemented as a wall boundary with a prescribed displacement profile. To this end, the membrane contour (*A*_Excor_) was extracted from a front view photograph of an inflated 30 mL Excor pump diaphragm using a tenth order polynomial fit function:

$$A_{\text{Excor}} = \mathop \sum \limits_{l = 0}^{10} p_{l} \cdot (r_{\text{Excor}} )^{l}$$

where *r*_Excor_ denotes the radial distance on the membrane (m), *p* the polynomial coefficients ($m^{{\left( {1 - l} \right)}}$) and $l$ the polynomial order (–).

Time-periodic membrane motion was programmed by superimposing this membrane contour with a sinusoidal time evolution of heart rate 75 bpm and 1:1 diastolic/systolic support mode:

$$z_{\text{Excor}} = A_{\text{Excor}} \cdot { \sin }\left( {\frac{2\pi t}{f}} \right)$$

where *t* denotes the time (s) and *f* the frequency (Hz).

Mesh morphing was employed for dynamic mesh adaptation to accommodate the membrane deflection.

The numerical solution of the Navier–Stokes equations was derived based on the Finite Volume Method, while turbulence was accounted for by implementing the Reynolds Averaged Navier–Stokes *k*–*ω* Shear Stress Transport turbulence model.^21^ The time step was set to 10^−3^ s while all residuals were required to decay below 10^−4^ to ensure convergence.^13^,^18^

Blood was modelled as Newtonian fluid with constant density *ρ* = 1050.0 kg m^−3^ and dynamic viscosity *μ* = 3.5 mPa s.^1^ Blood stagnation was assumed if flow velocities fell below 0.02 m s^−1^. To evaluate chamber washout, a passive scalar transport equation was implemented and, at the cannula inlet, a Dirichlet boundary condition was imposed on the passive scalar representing the dye concentration *ϑ* (–)^18^:

$${\text{Dye Washout}}:\,\,\vartheta = \left\{ {\begin{array}{ll} 1 &\quad if\, c = 1 \\ 0 &\quad else \\ \end{array}} \right.$$

where *c* is the cycle number. The dye was injected during one cycle, while subsequently being ejected over consecutive cycles. The duration *t*_95_ (s) served as a measure of time until 95% of the dye volume $\mathop \smallint \limits_{V}^{{}} \vartheta dV$ was washed out.

Further, a continuous arbitrary Lagrangian–Eulerian formulation of residence time was implemented and the time RT_1_ (s) employed as a metric for average blood residence time as proposed by Long *et al.*^18^:

$${\text{Residence Time}}:RT_{1} = \frac{1}{T \left| V \right|}\mathop \smallint \limits_{T}^{{}} \mathop \smallint \limits_{\varOmega }^{{}} H\left( \varvec{x} \right) \tau \left( {\varvec{x},t} \right) d\varOmega dT$$

$${\text{with}}\, \left| V \right| = \frac{1}{T}\mathop \smallint \limits_{T}^{{}} \mathop \smallint \limits_{\varOmega }^{{}} H\left( \varvec{x} \right)d\varOmega dT \quad{\text{and}}\quad H\left( \varvec{x} \right) = \left\{ {\begin{array}{ll} {1 } &\quad if\, x \in V \\ 0 &\quad else \\ \end{array}} \right.$$

where **x** denotes the spatial coordinates (m), *H*(**x**) the Heaviside function (–), *T* the cycle period (s), $\varOmega$ = V the instantaneous blood chamber volume including the cannula (mL), and *τ* the residence time distribution (s).

An objective function was defined to account for washout performance (t_95_, RT_1_) as previously introduced in a shape optimization study of a pulsatile ventricular assist device (pVAD),^19^ while additionally considering peak shear stresses and blood stagnation. Iterative hydraulic design optimization was performed to converge upon an appropriate pump configuration. Four design variables were studied including the pump chamber height *h* (m), the cannula diameter *d* (m), the cannula offset distance *w* (m) and the cannula orientation *θ* (°) (Figs. 1a–1d), each of which modified within a specified geometric range (Supplementary Table 1). Each design iteration was sequentially implemented and studied while evaluating the objective function to be minimized. This process converged towards a symmetric and an asymmetric design (Figs. 1a–1d) and the comparison thereof is described below.

### Validation of Numerical Simulations Using 4D-Flow MRI

To validate the numerical simulations, the manufactured symmetric and asymmetric prototypes (Figs. 1a–1d) were assessed using 4D-flow MRI performed on a 1.5T GE Discovery MR450 scanner (GE, Milwaukee, USA). The CoPulse pneumatic and control systems were housed outside of the scanner room (Fig. 2c). An 8 mm diameter airline was passed through the penetration panel to drive the pump within the scanner bore. The pump was submerged in a static water bath and connected *via* inflow cannula to a cylinder (diameter: 120 mm, height: 290 mm) filled with a glycerol/water mixture mimicking blood viscosity (3.0 mPa s). The cylinder acted as an open reservoir, while driving pressures for the pump were adapted to achieve physiologic filling/ejection timings and waveforms analog to previous *in silico* and *in vitro* results.^11^

A replica ECG signal was passed from the pump control unit to the scanner ECG input to allow MRI cardiac gating as per a typical ECG gated 4D-flow examination.^8^ A 4-point encoding 4D-flow sequence was acquired using a 32-channel phased-array cardiac coil covering the pump and surrounding water bath. A velocity encoding value of 160 cm s^−1^ was used based on earlier CFD simulations and confirmed during preliminary testing to avoid phase aliasing. For all acquisitions, data were acquired in a coronal orientation with an acquired isotropic resolution of 2.0 mm^3^, which was reconstructed to an inplane voxel size of 1.4 × 1.4 mm and slice thickness of 2.0 mm. Other 4D-flow MRI acquisition parameters were: echo time (2.46 ms), repetition time (4.42 ms), flip angle (7°), number of phases (25), and temporal resolution (32 ms). Signal averaging (NEX) of 4 was used. In addition to the 4D-flow sequence, a high temporal (20 ms) and spatial (0.94 × 0.94 mm inplane, 3.0 mm slice thickness) resolution Fiesta Cine sequence was acquired providing clear resolution of the pump membrane movement.

Static data from the water bath was manually segmented and used to correct for background phase artifacts by fitting a third order polynomial function.^28^ Post-processing of MRI data was performed using previously validated in-house software and Paraview (Kitware Inc., NY, USA).^6^

To produce CFD simulations comparable to the dynamics of the 4D-flow MRI data for validation, the sinusoidal membrane timing in the numerical setup was replaced by the actual diaphragm timing extrapolated from the MR scan. Validation was performed by comparing flow and velocity profiles from CFD and 4D-flow MRI data at probe points within the cannula and near the membrane insertion.

## Results

### Hemodynamic Evaluation

#### *In Vitro* Evaluation in a Hybrid Mock Circulation and Validation of *In Silico* Results

The mean hemodynamic values at each baseline and CoPulse support condition for the four different HFpEF phenotypes are shown in Table 1.

**Table 1:** Mean hemodynamic, pump, and energetic values from *in vitro* hybrid mock loop HFpEF phenotype experiments.

Hemodynamic parameter	HFpEF I			HFpEF II			HFpEF III			HFpEF IV		
	Baseline	CoPulse support	Change (%Change)	Baseline	CoPulse support	Change (%Change)	Baseline	CoPulse support	Change (%Change)	Baseline	CoPulse support	Change (%Change)
AoPmean (mmHg)	81	101	20 (25%)	75	90	15 (20%)	76	91	15 (20%)	93	105	12 (13%)
LAPmean (mmHg)	20	13	− 8 (− 27%)	19	13	− 7 (− 34%)	24	17	− 7 (− 28%)	26	18	− 8 (− 30%)
PAPmean (mmHg)	30	25	− 5 (− 16%)	28	24	− 4 (− 16%)	31	26	− 5 (− 16%)	36	30	− 6 (− 17%)
COmean (L/min)	3.2	4.2	1 (31%)	4.1	5.0	1 (22%)	4.1	5.0	1 (22%)	6.2	7.1	1 (14%)
Heart Rate (bpm)	75	75	0 (0%)	90	90	0 (0%)	70	70	0 (0%)	72	72	0 (0%)
SV_LV_ (mL)	44.1	28.9	− 15 (− 35%)	46.3	28.1	− 18 (− 39%)	59.8	44.4	− 15 (− 26%)	87.0	71.5	− 15 (− 18%)
SV_LV+Pump_ (mL)	44.1	57.3	13 (30%)	46.3	56.6	10 (22%)	59.8	73.1	13 (22%)	87.0	99.4	12 (14%)
SV_Pump_ (mL)	–	32.4	–	–	32.7	–	–	33.0	–	–	33.2	–
SW_LV_ (J)	0.40	0.33	− .07 (− 18%)	0.39	0.27	− .12 (− 31%)	0.50	0.44	− .06 (− 12%)	0.97	0.90	− .07 (− 7%)
PE_LV_ (J)	0.18	0.27	.09 (49%)	0.22	0.32	.10 (46%)	0.23	0.32	.10 (43%)	0.40	0.48	.08 (20%)
PVA_LV_ (J)	0.59	0.60	.02 (3%)	0.61	0.59	− .02 (− 3%)	0.73	0.76	.04 (5%)	1.37	1.37	.01 (0.6%)
*W*_Pump_ (J)	–	0.41	–	–	0.38	–	–	0.39	–	–	0.45	–
Pump volumetric efficiency	–	41%	–	–	31%	–	–	40%	–	–	37%	–
Pump work efficiency	–	83%	–	–	68%	–	–	85%	–	–	85%	–

Mean parameter values are shown at baseline and with CoPulse pump support for each HFpEF phenotype, along with the absolute changes and % changes in parentheses of the hemodynamic parameter values from baseline to CoPulse pump support. HFpEF I denotes the phenotype of genetically inherited hypertrophic cardiomyopathy, HFpEF II the state of infiltrative cardiomyopathy with restrictive physiology, HFpEF III the condition of nonhypertrophic cardiomyopathy without significant cardiac comorbidities, while HFpEF IV refers to patients with normal ejection fraction with significant comorbidities (i.e. hypertension, coronary artery disease), and typically, hypertrophy.^5^

Baseline unsupported conditions were indicative of typical HFpEF hemodynamics, such as elevated LAP (> 19 mmHg) and high PAP (> 28 mmHg). CoPulse pump support decreased mean LAP (27–34%) and mean PAP (16–17%) while increasing mean AoP (13–20%) and mean CO (14–31%). With a SV_Pump_ of 32.4–33.2 mL, the ${\text{SV}}_{\text{LV}}$ was reduced by 15–18 mL (18–39%), but the overall ${\text{SV}}_{{{\text{LV}} + {\text{Pump}}}}$ increased by 10–13 mL (14–30%). This equated to a 31–41% pump volumetric efficiency and 68–85% pump work efficiency. The lowest efficiencies were observed for the HFpEF II phenotype.

By filling synchronously during LV filling and ejecting during LV ejection, the pump performs 0.38–0.45 J of hydraulic work as it unloads the LV, and consequently, the LA during diastole, and ejects volume into the LV and systemic circulation during systole. This resulted in a reduction in SW_LV_ of .06–.12 J (7–31%) and an increase in PE_LV_ of .08–.10 J (20–49%) for an overall small change in PVA_LV_ of .02–.04 J (3–5%).

Comparing mean hemodynamic values to those obtained from previously conducted *in silico* results,^11^ errors for AoP, LAP, and PAP were less than 1.3 mmHg (< 3.98%), and errors for CO were less than .081L min^−1^ (< 1.33%). PV loops of the LV from simulations and the *in vitro* experiments are displayed in Supplementary Figure 3 and show a high level of agreement (RMSE < 2.8 mmHg between LVP_simulation_ and LVP_*in vitro*_, RMSE < 2.0 mL between LVV_simulation_ and LVV_*in vitro*_).

#### *Ex Vivo* Evaluation in Isolated Heart Experiments

**Figure 3:**
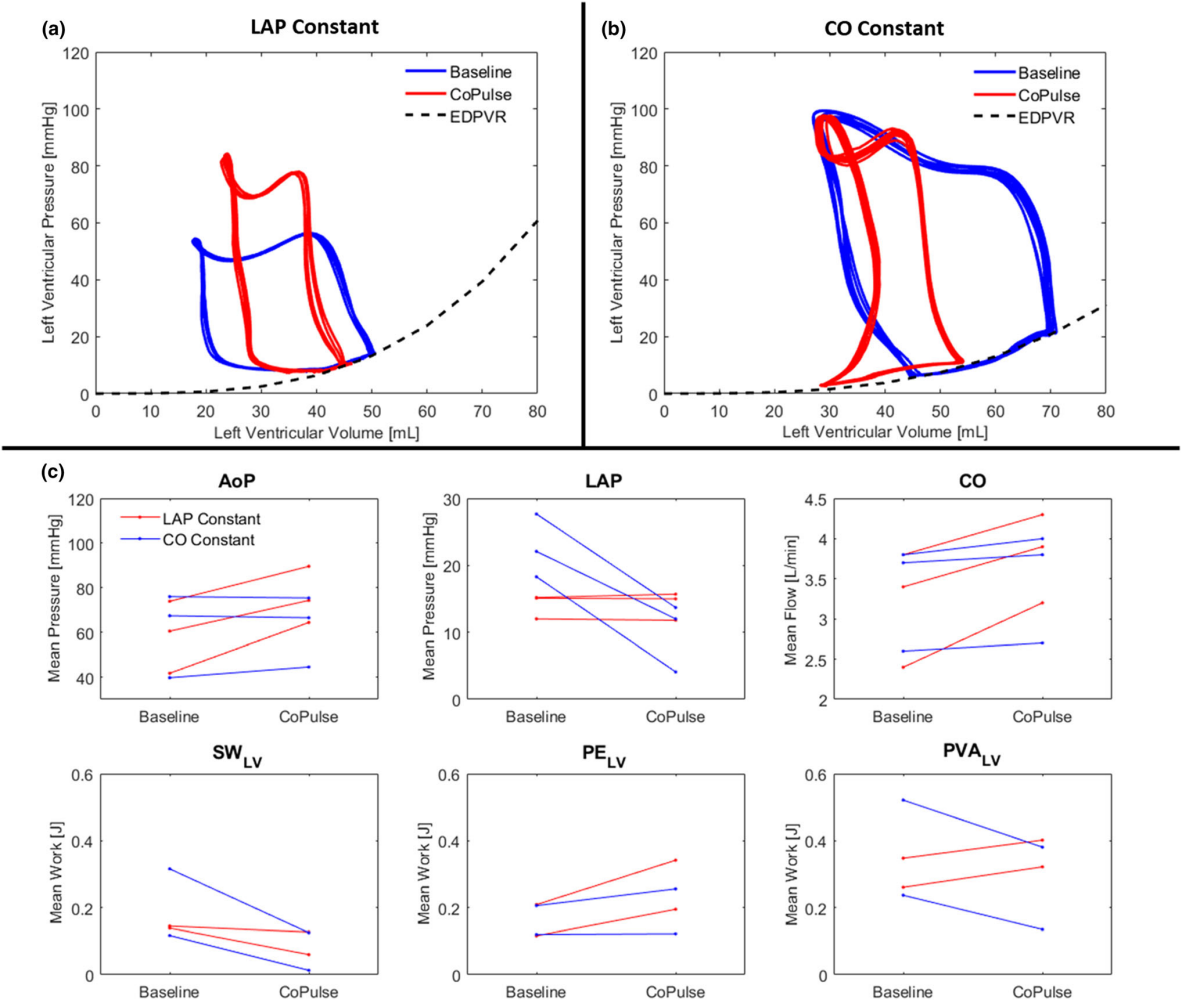
Representative PV loops at baseline and pump support conditions during *ex vivo* isolated heart constant LAP and constant CO experiments are shown in (a) and (b), respectively. When LAP was held constant, ESV was noticeably increased. When CO was held constant, the ESV was not noticeably affected. Mean hemodynamic values at baseline and pump support conditions during all *ex vivo* experiments are shown in (c).

In constant LAP experiments, preload was maintained (LAP changed 0.3 ± 2.2%) at a constant heart rate (HR changed 1.2 ± 3.7%) between baseline and CoPulse support conditions (Fig. 3c). All preload experiments exhibited increased AoP (33 ± 15%) and CO (21 ± 9.3%) with CoPulse support. Additionally, CoPulse support decreased SW_LV_ (35 ± 22%), and increased PE_LV_ (65 ± 1.9%) for an overall increase in PVA_LV_ (19 ± 3.7%). The minimum ESV was increased from 17.6 mL to 22.7 mL during CoPulse support (Fig. 3a).

In constant CO experiments, CO was maintained (CO changed 3.1 ± 0.5%) at a constant heart rate (HR changed 0.3 ± 0.5%) between baseline and CoPulse support conditions (Fig. 3c). All CO experiments exhibited decreased LAP (58 ± 14%) with CoPulse support. Additionally, CoPulse support decreased SW_LV_ (75 ± 14%) with greater magnitude than it increased PE_LV_ (10 ± 9.8%) for an overall decrease in PVA_LV_ of 36 ± 7.4%. The minimum ESV increased from 27.0 mL to 28.3 mL during CoPulse support (Fig. 3b).

### Fluid Dynamic Evaluation

#### Numerical Simulations

**Figure 4:**
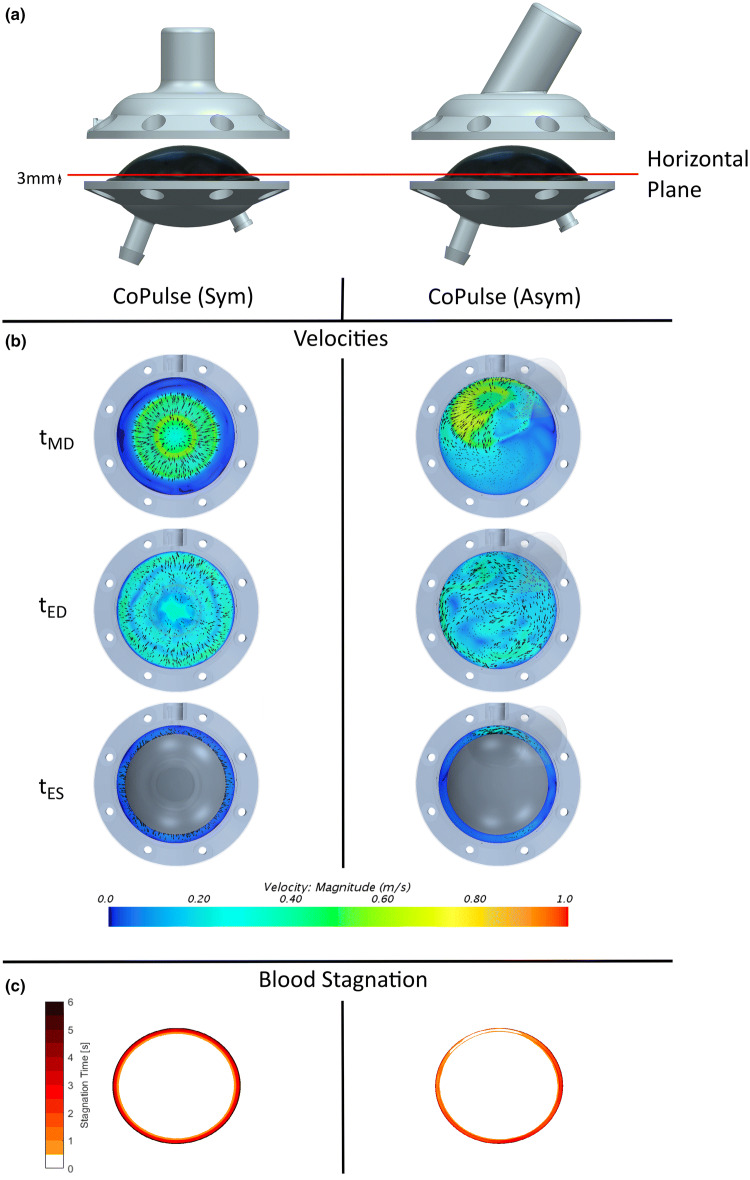
(a) Location of the horizontal analysis plane in the symmetric and the asymmetric prototype. To provide visibility of the Excor membrane and the horizontal analysis plane, the housing of the blood chamber is lifted. (b) Velocity fields in horizontal analysis plane for the symmetric and asymmetric design of the CoPulse pump at the time instants mid-diastole (*t*_MD_), end-diastole (*t*_ED_) and end-systole (*t*_ES_). (c) Temporal distribution of stagnation zones illustrated for the symmetric and the asymmetric design of the CoPulse pump.

Flow distribution was analyzed on a horizontal analysis plane (Fig. 4a). The flow field in the outermost region of the symmetric CoPulse configuration (Fig. 4b, left) revealed velocity magnitudes below the stagnation threshold throughout mid-diastole (*t*_MD_), end-diastole (*t*_ED_), and end-systole (*t*_ES_). In contrast, the asymmetric cannula configuration (*w* = 18 mm, *θ* = 60°) introduced substantial azimuthal, unidirectional flow (> 0.02 m s^−1^) that perfused the pump circumference throughout the entire pump cycle (Fig. 4b, right). Consequently, areas of prolonged blood stagnation exceeding 5 s in a simulation of six consecutive pump cycles were significantly reduced by 74.35% (8.06 vs. 31.42 mm^2^) in the asymmetric design as compared to its symmetric counterpart (Fig. 4c).

**Figure 5:**
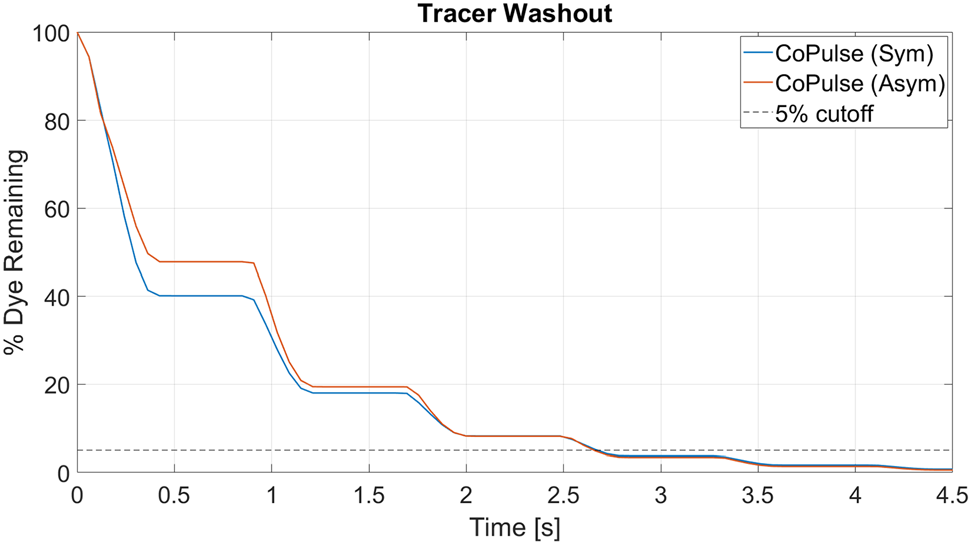
Time evolution for dye washout in the symmetric and the asymmetric design of the CoPulse pump. The virtual dye injection experiment started with the systolic phase (*t* = 0–0.4 s) where some of the tracer was expelled together with blood, causing a drop in the dye concentration. In the subsequent filling phase (*t* = 0.4–0.8 s), pure blood entered the pump while the tracer concentration within the chamber remained constant. This cycle recurred throughout the entire pumping process, eventually resulting in a staggered progression of dye washout.

The time evolution of dye washout in both pump designs is illustrated in Fig. 5. After a first pump cycle (*t* = 0–0.8 s), less dye was remaining in the symmetric configuration (40.15%) as compared to its asymmetric counterpart (47.79%). However, in the subsequent cycles, similar washout characteristics with comparable values in *t*_95_ (2.67 s vs. 2.66 s) and RT_1_ (0.99 s vs. 0.95 s) were observed in both designs of the CoPulse pump.

In both configurations, peak wall shear stresses occurred during ejection at the site where the cannula attaches to the pump chamber (symmetric design: 15.65 Pa, asymmetric design: 23.56 Pa). Peak shear stresses on the membrane occurred during filling at the site of the entering jet (symmetric design: 10.80 Pa, asymmetric design: 18.11 Pa).

#### Validation of Numerical Simulations

**Figure 6:**
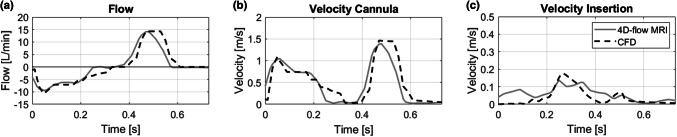
Comparison of flow (a) and velocity profiles (b, c) among 4D-flow MRI and CFD for the symmetric design of the CoPulse pump. The profiles are represented for a single pump cycle, starting with diastole followed by systole.

Flow and velocity profiles of CFD and 4D-flow MRI data are depicted in Fig. 6. The profiles illustrate the time course of a single pump cycle including diastole (*t* = 0–0.25 s), end-diastolic period (*t* = 0.25–0.36 s), systole (*t* = 0.36–0.59 s), and end-systolic period (*t* = 0.59–0.8 s).

CFD flow and velocity profiles in the cannula showed good correlation (*r* > 0.94) with the profiles measured by 4D-flow MRI (Figs. 6a and 6b). Near the membrane insertion (Fig. 6c), the simulation produced more conservative results with velocity magnitudes well below values recorded in the MR measurements (*r* = 0.76). Yet, peak values were elevated 27.9% in the velocity computations compared to the MR scan.

**Figure 7:**
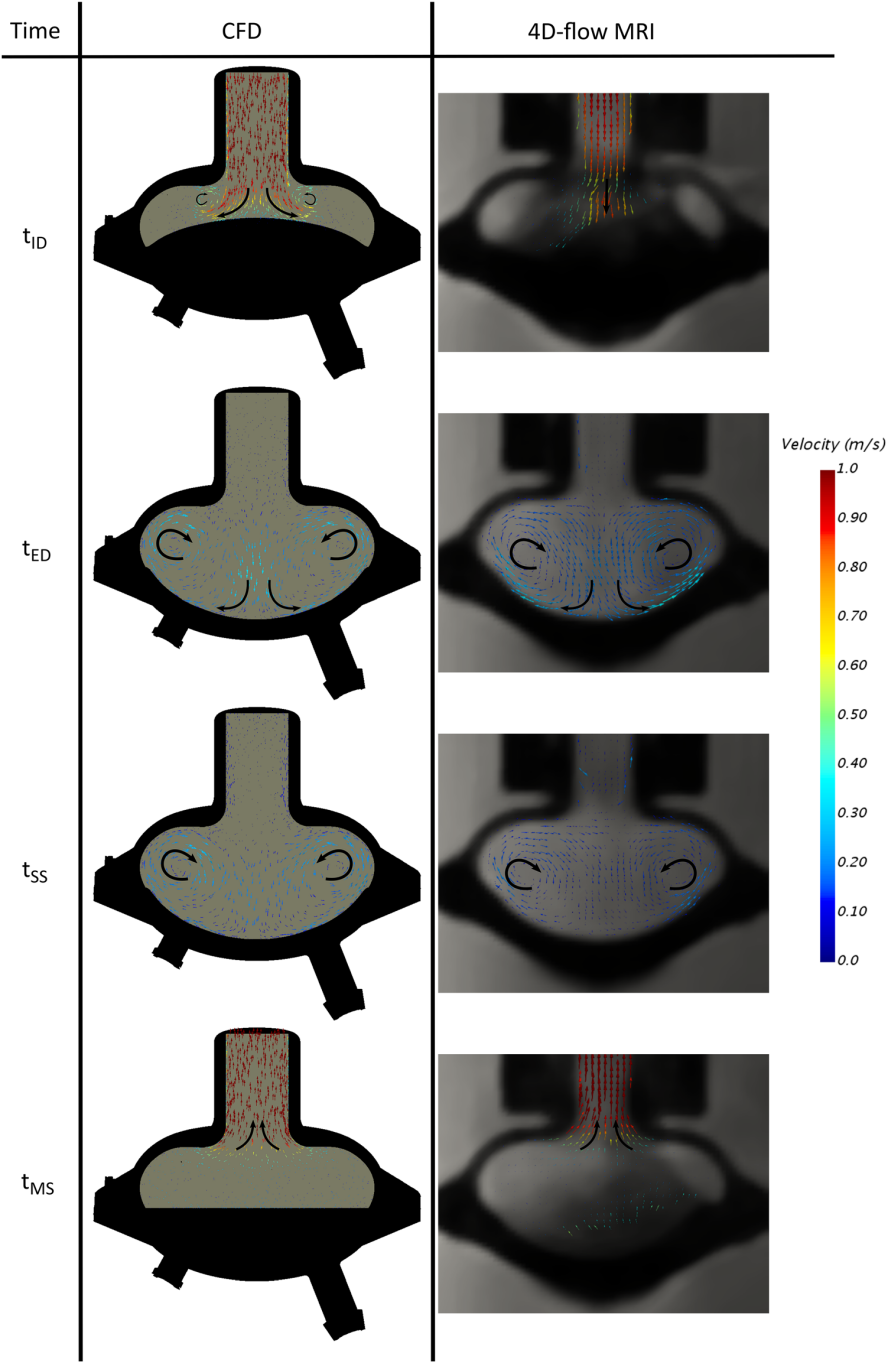
Comparison of CFD data (left) to 4D-flow MRI recording (right) for the symmetric design of the CoPulse pump. Illustration for the time instants initial-diastole (*t*_ID_), end-diastole (*t*_ED_), start of systole (*t*_SS_), and mid-systole (*t*_MS_)*—*cross sectional view.

The flow solution on a cross-section of the symmetric prototype from both the CFD simulation and the 4D-flow MRI are displayed in Fig. 7. The pneumatic port, which was not centrally positioned (Figs. 1b and 1e), led to the diaphragm deforming asymmetrically as evidenced by the MRI data (Fig. 7a right). At initial diastole (*t*_ID_), CFD and MRI data revealed comparable velocity distributions within the cannula (0.916 vs. 0.922 m s^−1^). The simplified membrane deflection in the numerical setup resulted in early axisymmetric vortex formation. In contrast, the actual asymmetric deformation apparent in the MR scan led to delayed vortex generation due to the rapid downward motion of the membrane tip. At end-diastole (*t*_ED_) and at the start of systole (*t*_SS_), vortices with similar flow features crystallized in both data sets, but with velocities appearing qualitatively elevated in the CFD computations. During mid-systole (*t*_MS_), the MR scan again disclosed asymmetric membrane deformation, however, with comparable flow patterns evident among CFD and MR results.

## Discussion

In this study, the development of the CoPulse pump—a valveless pulsatile MCS device—was progressed from a concept to a functional prototype. Previous numerical hemodynamic findings^11^ were validated *in vitro* and further substantiated *ex vivo*, and hemocompatible design feasibility was analyzed by combining CFD and 4D-flow MRI.

### Hemodynamic Evaluation

The hybrid *in vitro* test bench enabled testing of the physical CoPulse pump, pneumatic driver, and control system. The *in silico* results, which exhibited the pump’s potential to normalize hemodynamics of various HFpEF phenotypes,^11^ could be replicated with a marginal difference in mean hemodynamic findings (AoP, LAP, CO, PAP) of < 3.98% and close agreement in LVP and LVV. These results confirmed that the CoPulse pump may increase CO and reduce LAP—effects that could be especially valuable for HFpEF patients that are burdened by abnormally high filling pressures and compromised CO.

The *ex vivo* isolated heart provided a physical model of a heart in combination with the pump system to validate the interaction between the pump and the ventricle under controlled hemodynamic conditions. The hemodynamics and PV loops displayed the same trends and morphology as *in silico* and *in vitro* results. CoPulse pump support increased CO and MAP when the LAP was kept constant. In the case of constant CO, the LAP was reduced. In both cases, the ESV was not further reduced compared to the unsupported condition since the CoPulse pump only fills while the mitral valve is open. These results further confirmed the CoPulse’s hemodynamic effect and suggest that ventricles supported with the CoPulse pump may mitigate the risk of suction events, which constitutes a major obstacle for the treatment of HFpEF patients with MCS devices.^22^

Under the implemented mode of operation of the pump, volumetric efficiencies of 31–41% and pump work efficiencies of about 68–85% were observed. The volumetric efficiency can be interpreted as the percentage of SV_pump_ that is converted into useful CO. Part of the SV_pump_ is absorbed by the LV which primarily varies according to the systolic properties of the heart. This highlights the requirement of a stiff ESPVR for efficient functionality of the CoPulse pump. Another part of the SV_pump_ is converted into pressure within the LV, which is captured by the work efficiency of the pump. The work efficiency provided a measure of the total additional SW that is performed by the LV and pump compared to the unsupported HFpEF ventricle. Therefore, approximately 40% of the SV_pump_ is converted into CO while approximately 80% of the pump work is retained as useful SW of the LV and pump. The lowest volumetric and work efficiency was observed for HFpEF II as it represents the phenotype with the least stiff systolic properties and with relatively less-stiff diastolic properties. This causes the LV to absorb more energy during pump ejection and filling. Further studies are required to delineate the specific systolic and diastolic cardiac properties that optimally capitalize on the clinically relevant benefits of the CoPulse pump.

*In vitro* and *ex vivo* results showed that CoPulse support reduced SW_LV_ in exchange for increased PE_LV_ with nearly conserving the total PVA_LV_—a measure of the total mechanical energy consumption of the LV during a cardiac cycle. A similar exploration on the effect of synchronized MCS device support on LV energetics was conducted also with a single cannula pump, except in an chronic ovine model of dilated heart failure^15^ and an acute healthy ovine model.^16^ The study presented contrasting results that such a pump would increase overall SW_LV_ while still increasing CO by augmenting the total SV. This discrepancy can be attributed to the different modus operandi of pump ejection/filling phasing employed. Landesberg *et al*. fills the pump during isovolumetric relaxation, reducing the ESV and lowering the diastolic pressure in the ventricle prior to the beginning of ventricular diastole. This could result in an increased risk for suction events and increases the work performed by the diseased LV. By filling only during LV filling, the majority of the work performed during pump filling is on the circulatory system instead of the LV while still providing similar CO augmentation (22.5^16^ vs. 14–31% *in vitro* and 21 ± 9.3% *ex vivo*). Which modus operandi would better promote chronic reversal of underlying hypertrophy that contributes toward certain HFpEF phenotypes is debatable and demands long-term studies.

### Fluid Dynamic Evaluation

The fluid dynamic analysis disclosed an asymmetric pump configuration with an offset tilted cannula to provide the best trade-off among blood stagnation prevention, blood washout performance, and low levels of shear stresses. A similar design was previously introduced for a 30 mL single port valveless counter-pulsation device (Symphony, Abiomed Inc., MA, USA) that was to be connected to the subclavian artery.^9^ In a study combining CFD, particle image velocimetry (PIV), and animal experiments, the authors claimed the Symphony to be hemocompatible with low risks for hemolysis or thrombus formation and concluded the design to be adequate for human implantation. Yet, the pump dimensions deviate from those specified for the CoPulse pump and the narrow anatomical constraints of the pericardial space could impede implantability of the Symphony pump into the LV apex due to its tangential cannula configuration (*θ* = 0°).

To allow for good fit within the pericardial space when implanted into the LV apex, the asymmetric CoPulse pump incorporates a cannula attached at an angle of *θ* = 60°. This configuration proved sufficient to generate a persistent, unidirectional flow throughout the entire pump cycle, thus significantly reducing blood stagnation, and consequently, the risk for thrombus formation in the vicinity of the membrane insertion. However, the optimized asymmetric design revealed elevated peak shear stresses at the housing and on the membrane as compared to the symmetric design. The latter observation coincided with the findings of Xu *et al*.^29^ claiming the reduced potential for thrombus deposition to generally be at the expense of elevated risks for hemolysis or platelet activation. However, levels of shear stresses within the asymmetric CoPulse pump were significantly lower than those observed in the Symphony device^9^ and remained well below the threshold for platelet activation (50 Pa) or hemolysis (150 Pa).

The duration for 95% dye washout (*t*_95_ = 2.66 s) as well as the average blood residence time (RT_1_ = 0.95 s) calculated for the asymmetric CoPulse pump revealed slightly elevated values as compared to those previously reported for a pVAD with separate in- and outlet port (*t*_95_ = 2.47 s, RT_1_ = 0.893 s).^18^ However, the latter study was conducted for a 73 mL pump operated at 80 bpm, thus limiting direct comparison with the CoPulse pump (30 mL, 75 bpm). We performed a sensitivity analysis that, along with the publication of Xu *et al*.,^29^ indicated the elevated heart rate and stroke volume in the adult pVAD to be in favor of good washout as compared to the CoPulse pump. Increasing the stroke volume of the CoPulse pump to 35 mL already resulted in comparable dye washout (*t*_95_ = 2.55 s) and superior blood residence time (RT_1_ = 0.835 s) as compared to the adult pVAD. This further demonstrates the feasibility of a single cannula valveless design with comparable hemocompatibility features inherent to conventional pVADs with separate in- and outflow cannula that are commonly implanted in HFrEF patients.

CFD-based methodologies, as presented in this study, are widely accepted as a powerful tool for flow inspection and hemocompatibility characterization within VADs. However, the validation of such numerical studies is of paramount importance to assure reliable results. For this purpose, CFD data is commonly compared against flow visualization experiments.^1^ Within the frame of this study, 4D-flow MRI was employed for CFD-validation. The recordings revealed the membrane to deflect asymmetrically mainly due to the offset pneumatic port. Despite the idealized membrane motion implemented in the CFD setup, the results showed a high level of agreement among CFD and MRI data (*r* > 0.76), wherefore validity of the CFD results was assumed.

## Limitations

Although the developed models are fit for investigating the acute effects of the pump, they do not account for the influences of some physiological adaptive mechanisms (e.g. neural, hormonal). Without the establishment of an adequate large animal model of HFpEF, the *in-vivo* evaluation for such a device is challenging. The isolated heart model has previously been used for evaluation of other MCS devices^10^ and is particularly adapted for controlled hemodynamic and visual investigations. The isolated hearts indicated decreased diastolic compliance, a common characteristic of HFpEF phenotypes, made evident by the steep EDPVR found for all the porcine hearts; however, ESPVR was flatter for some porcine hearts additionally indicating reduced systolic function (Fig. 3b).

The hydraulic design of the CoPulse pump was optimized at a typical operating point of 75 bpm and under the assumption of a simplified membrane motion. Although the numerical simulation did not accurately capture the actual membrane motion, CFD data disclosed good agreement with 4D-flow MRI. However, the impact of the actual asymmetric membrane motion on the fluid dynamics within the pump chamber remains to be studied under a broader range of operating settings in order to assure favorable hemocompatibility characteristics for all clinically relevant conditions. For this purpose, fluid-structure-interaction simulations are proposed to accurately capture the realistic membrane motion by considering the interaction of air, blood and diaphragm at relevant operating conditions. To validate those *in silico* hemocompatibility characteristics, *in vitro* and *in-vivo* studies are to be conducted in order to claim appropriate pump hemocompatibility.

## Conclusion

The *in vitro* hybrid mock circulatory loop and the *ex vivo* isolated porcine heart model disclosed the CoPulse pump to improve hemodynamics for specific HFpEF phenotypes by lowering LAP and increasing CO while potentially obviating the risk for suction events. Further, hemocompatible design feasibility was demonstrated, revealing moderate shear stresses with comparable characteristics for pump washout and blood residence time as previously reported for a pVAD with two cannulas typically implanted in HFrEF patients.^18^ This successful completion of the early stage device development process supports the further progression of the CoPulse pump.

## Electronic supplementary material

Below is the link to the electronic supplementary material.

Supplementary material 1 (TIFF 8258 kb)

Supplementary material 2 (TIFF 2252 kb)

Supplementary material 3 (TIFF 154 kb)

Supplementary material 4 (DOCX 13 kb)

## Abbreviations

HF: Heart failure
EF: Ejection fraction
HFrEF: Heart failure with reduced ejection fraction
HFmrEF: Heart failure with mid-range ejection fraction
HFpEF: Heart failure with preserved ejection fraction
LAP: Left atrial pressure
MCS: Mechanical circulatory support
LV: Left ventricle
SV: Stroke volume
VAD: Ventricular assist device
LVP: Left ventricular pressure
LVR: Left ventricular reservoir
AoP: Aortic pressure
PAP: Pulmonary artery pressure
CO: Cardiac output
LVV: Left ventricular volume
PR: Preload reservoir
AoR: Aortic reservoir
VR: Venous reservoir
AoF: Aortic flow
CoF: Coronary flow
PV: Pressure-volume
EDPVR: End-diastolic pressure volume relationship
EDV: End-diastolic volume
ESV: End-systolic volume
HR: Mean heart rate
SW_LV_: Left ventricular stroke work
PE_LV_: Left ventricular potential energy
PVA_LV_: Left ventricular pressure-volume area
ESPVR: End-systolic pressure volume relationship
CFD: Computational fluid dynamics
MRI: Magnetic resonance imaging
ECG: Electrocardiography
RMSE: Root mean square error
PIV: Particle image velocimetry
pVAD: Pulsatile ventricular assist device
