# Supplementary figures and images for: A Valveless Pulsatile Pump for Heart Failure with Preserved Ejection Fraction: Hemo- and Fluid Dynamic Feasibility
Source: Ann Biomed Eng. 2020 Mar 30;48(6):1821–36. doi: 10.1007/s10439-020-02492-2 (PMC7280352; doi:10.1007/s10439-020-02492-2)

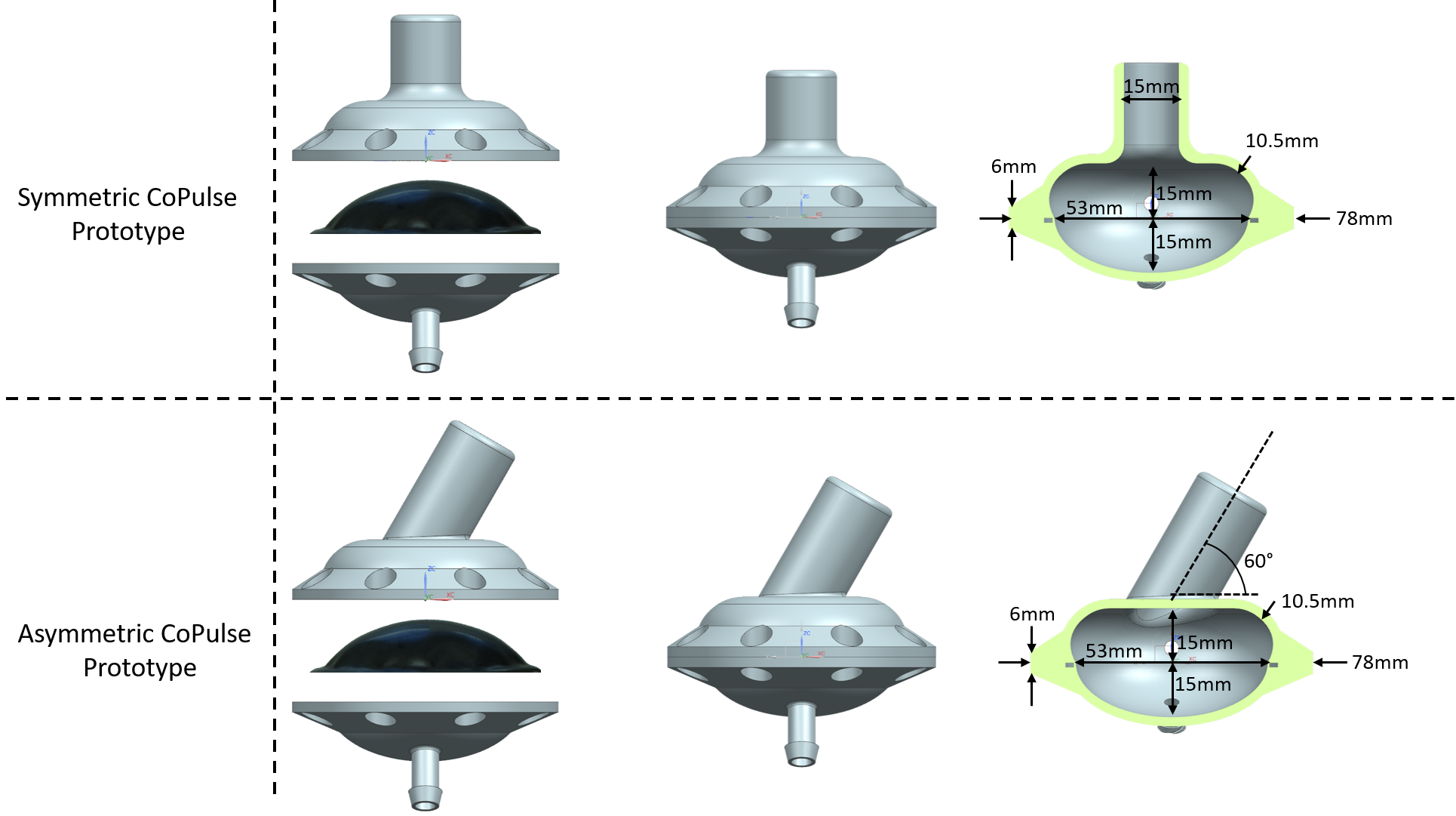

Supplement: Supplementary file 1 — Supplementary material 1 (TIFF 8258 kb) [file 10439_2020_2492_MOESM1_ESM.tiff]

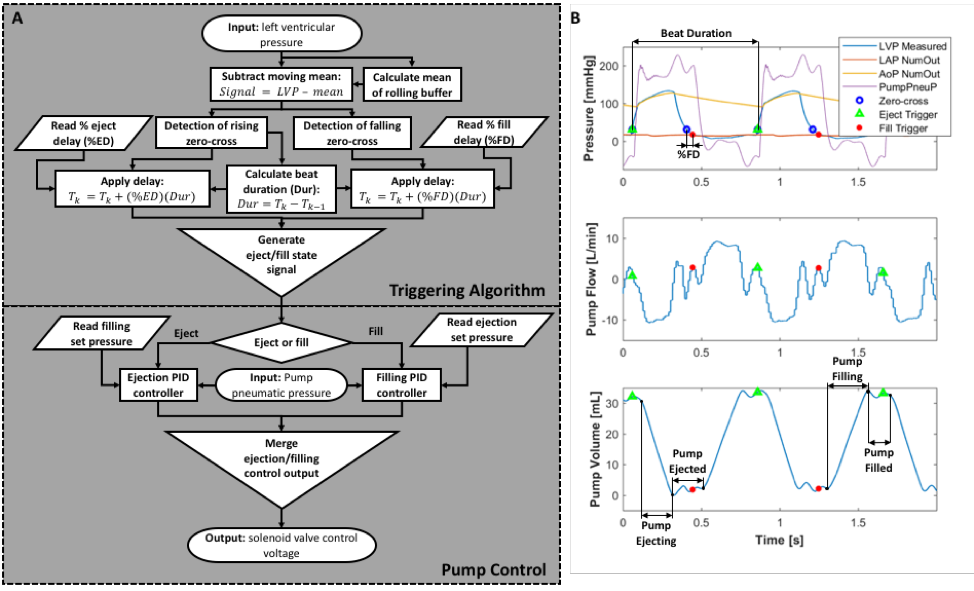

Supplement: Supplementary file 2 — Supplementary material 2 (TIFF 2252 kb) [file 10439_2020_2492_MOESM2_ESM.tiff]

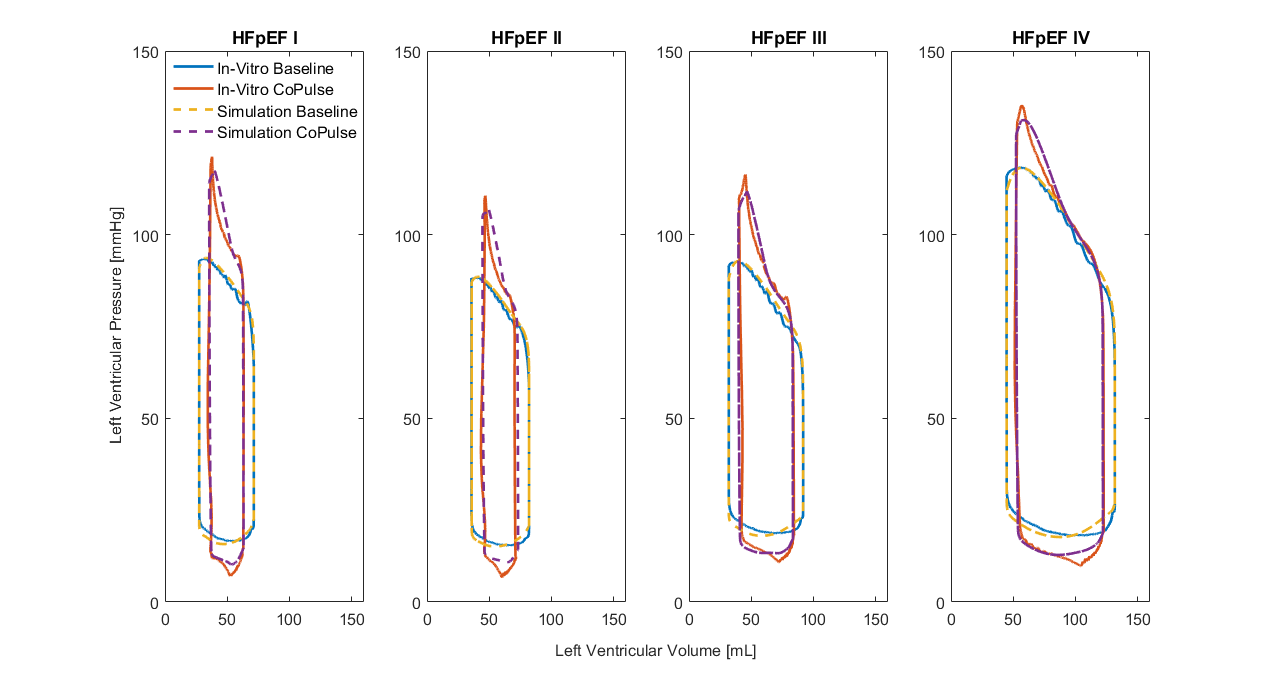

Supplement: Supplementary file 3 — Supplementary material 3 (TIFF 154 kb) [file 10439_2020_2492_MOESM3_ESM.tif]
