# Supplementary material for: A Valveless Pulsatile Pump for Heart Failure with Preserved Ejection Fraction: Hemo- and Fluid Dynamic Feasibility
Source: Ann Biomed Eng. 2020 Mar 30;48(6):1821–36. doi: 10.1007/s10439-020-02492-2 (PMC7280352; doi:10.1007/s10439-020-02492-2)
Supplement: Supplementary file 4 — Supplementary material 4 (DOCX 13 kb) [file 10439_2020_2492_MOESM4_ESM.docx]

Design variables and their respective geometric range employed for the iterative design optimization

| **Design Variable** | **Symbol** | **Geometric Range** |
| --- | --- | --- |
| Pump Chamber Height | h | 25mm, 30mm, 35mm |
| Cannula Diameter | d | 10mm, 15mm |
| Cannula Offset Distance | w | 0mm, 10mm, 22mm |
| Cannula Orientation (Roll Angle) | θ | 60°, 90° |

Supplementary Table 1 – The four design variables h (pump chamber height), d (cannula diameter), w (cannula offset distance), and θ (cannula orientation) employed for the hydraulic optimization of the CoPulse pump with their respective geometric range.
